# Remote anchorage implants as an emerging treatment modality for the severely atrophic maxilla

**DOI:** 10.34172/japid.026.4247

**Published:** 2026-01-05

**Authors:** Mohammadreza Talebi Ardakani, Aida Kheiri

**Affiliations:** ^1^Department of Periodontics, School of Dentistry, Shahid Beheshti University of Medical Sciences, Tehran, Iran; ^2^Dental Research Center, Research Institute of Dental Sciences, Shahid Beheshti University of Medical Sciences, Tehran, Iran

## To the Editor,

 Dental implant placement is a well-known treatment approach with predictable outcomes. However, severe maxillary atrophic ridges caused by periodontitis, trauma, malignancies, and developmental problems can notably alter the treatment course.^1,2^ In the absence of natural teeth, maxillary sinus pneumatization and alveolar bone resorption compromise the available bone for ideal implant placement. Over the years, conventional treatment modalities for the rehabilitation of edentulous maxilla have been investigated. Maxillary sinus lift surgery, onlay bone grafting, ridge-splitting techniques, and interpositional bone grafting are among these treatment options that carry multiple limitations, including patient morbidity, infection risk, higher costs, and longer treatment periods. To overcome these drawbacks, remote anchorage sites were introduced as a more straightforward approach.^3-5^ Remote anchorage implantology is the strategic use of distant cortical bone in atrophic jaws, offering graft-free treatment while enhancing implant stability and load distribution.^3^

 First developed, zygomatic implants were introduced by Brånemark’s team in 1998 to support large maxillofacial prostheses in patients who underwent maxillectomies.^5^ Over the years, by considering anatomic considerations, degree of bone loss, and bone density, several classifications of atrophic maxilla were developed. By applying these classifications, other suitable extra-alveolar sites, as well as proper implant designs, including transnasal, tuberosity, transsinus, and pterygoid implants, were suggested.^1,3^

 Brånemark’sprotocol, quad-zygoma approach, pterygoid/nasal cortex implants, and a combination of all these alternatives have been introduced to rehabilitate a partial or complete edentulous atrophic maxilla.^3^ These treatment options have provided a superior anterior-posterior spread (A-P spread) and reduced or even eliminated the length of distal cantilevers of the final fixed prosthesis. Pooled data from a recent systematic review also showed a considerable cumulative survival rate of 95.5% of pterygoid implants in a 6-year follow-up.^6^ Moreover, the possibility of immediate loading, only a few hours after the surgery, offers higher patient-reported outcomes like aesthetic, function, and overall satisfaction (PROMs).^7^

 However, grey zones in these modalities still need to be addressed. Proper implant designs, from macro- and microstructural perspectives, can significantly affect implant positioning and surgical procedures. On the other hand, according to a radiographic-based study,^8^ anatomic variations, including foramina and canals within the zygomatic bone or other anatomic markers at other anchorage sites, should be clearly identified to prevent surgical complications.

 In patient selection, relative and absolute contraindications are similar to those of conventional implants.^9^ Nonetheless, extra-alveolar implant placement requires experienced and expert surgeons since defining the exact point for implants’ exit, cortical stabilization achievement, limited mouth opening in the posterior area, and invasive flap management can be difficult.^7,9^

 From a critical perspective, though remote anchorage implants can result in significant improvements in patients’ quality of life, they may entail certain drawbacks that should be noted. Possible complications include biomechanical stress, paresthesia, sinusitis, orbital penetration, and hematoma.^3^ Since one of the primary evaluated outcomes is patient-related parameters, patients should be informed regarding any of these possible complications.

 It is often recommended in complex cases that these surgeries be performed under general anesthesia for better patient compliance and comfort. During the surgical procedure, local anesthesia with a vasoconstrictor is recommended to control local bleeding and postoperative pain and discomfort.^10^ To overcome the aforementioned technical and anatomical struggles, surgeons would benefit from performing a customized surgical plan for each patient.^7^ Presurgical prosthetic planning, 3D imaging analysis, application of surgical guides, and navigation systems can considerably impact surgical outcomes.^7,10^

 Despite the emergence of remote anchorage implants as a viable treatment option for patients with severely atrophic maxillae, there are limited high-quality prospective data on the precise success criteria, long-term reported survival rates in multicenter studies, and the management of soft and hard tissues. Therefore, future investigations addressing these unresolved questions may clarify treatment selection in severely atrophic maxillae.

## Competing Interests

 The authors declare that they have no competing interests.
